# MPFL reconstruction and tibial tuberosity transposition in patients with patellar instability: May it troubleshots also trochlear dysplasia?

**DOI:** 10.1186/s40634-021-00392-5

**Published:** 2021-10-30

**Authors:** A. Castelli, E. Jannelli, E. Ferranti Calderoni, G. Galanzino, A. Ivone, L. Caliogna, C. Klersy, G. Pasta, M. Mosconi, F. Benazzo, G. Zanon

**Affiliations:** 1 Department of Orthopaedic and Traumatology Surgery, Fondazione IRCCS Policlinico San Matteo, Pavia, Italy; 2 Department of Orthopaedic and Traumatology Surgery, Ospedale Maggiore “Carlo Alberto Pizzardi”, Bologna, Italy; 3 Biometrie and Clinical Epidemiology Service, Fondazione IRCCS Policlinico San Matteo, 27100 Pavia, Italy

## Abstract

**Purpose:**

This study aimed to highlight short- and medium-term outcomes of combined medial patello-femoral ligament (MPFL) reconstruction and anterior tibial tuberosity (ATT) transposition surgery in patients with recurrent patellar instability and different degrees of trochlear dysplasia.

**Methods:**

Between January 2014 and May 2019, 25 patients with patellar instability underwent a surgical procedure combining the lowering/transposition of the ATT and the MPFL reconstruction. Each patient were preoperative assessed by Kujala score, International Knee Documentation Committee (IKDC), Tegner activity level scale. The assessment of instability predisposing factors was carried out with patellar height, tibial tuberosity-trochlear groove (TT-TG) distance, trochlear dysplasia, sulcus angle, patellar tilt and MPFL injuries. Functional outcomes were evaluated with Kujala, IKDC and Tegner scores at 3, 6 and 12 months after surgery.

**Results:**

The average age of the patients was 20 years (range 13–43 years). Pre- operative Caton–Deschamps index was pathological in 10 (40%). Sulcus angle was elevated in 13 patients (52%) and TT-TG distance was irregular in 17 patients (68%). Trochlear dysplasia was present in 13 patients (9 type A, 3 type B, 1 type C according to Dejour’s Classification). No re-dislocation occurred during the follow-up. There was a significant increase in the Kujala, IKDC and Lysholm scores after 3, 6 and 12 months, and the results were compared for the different follow-up times and patient’s trochlear dysplasia degree.

**Conclusion:**

This prospective observational longitudinal study identified good clinical outcomes in patients who underwent MPFL reconstruction and ATT transposition for patellar instability. Finally, the different risk factors for patellar instability examined, particularly the presence of trochlear dysplasia, did not significantly influence the final functional results, which range from good to excellent without re-dislocation episodes.

## Introduction

Recurrent patellar instability is quite common in young and active people. Without appropriate treatment, these injuries may result in significant morbidity, including osteochondral fractures, limitations in daily and sport activity, and patellofemoral arthritis [31]. Trochlear dysplasia is one of the most important predisposing factors, having been confirmed as a radiological finding in 85% of patients with a history of recurrent lateral patella dislocations [2]. Other important bony abnormalities that contribute to patellar instability are high tibial tubercle to trochlear groove (TT-TG) distance, patellar height, pathological patellar tilt and sulcus angle [1, 8]. Moreover, medial patellofemoral ligament (MPFL) rupture, which contributes from 50 to 60% of the total medial restraining force against lateral patellar dislocation at 0° to 30° degrees of knee flexion [7], represents the main risk factor among all soft-tissue abnormalities that cause patellar instability. In the last decades, MPFL reconstruction concomitant with anterior tibial tuberosity (ATT) transposition has become more and more popular due to the better subjective clinical outcomes and lower re-dislocation rate. Isolated MPFL reconstruction and additional soft-tissue procedures is adopted in the case of failure in patients with dysplastic trochlea, patella alta, and/or elevated TT-TG distance [24]. Good clinical results have also been achieved with combined surgical treatment in patients with a high degree of trochlear dysplasia [2]. This surgical treatment gave an effective solution also in patients who might require trochleoplasty, which is an invasive surgery, without a long follow-up of outcomes and with a high risk of patellofemoral arthritis [4]. This study aimed to highlight short- and medium-term outcomes of combined MPFL reconstruction and ATT transposition surgery in patients with recurrent patellar instability. Our hypothesis was that patients with recurrent patellar instability could achieve good clinical outcomes and low re-dislocation rate with this combined surgical treatment, in spite of other anatomic abnormalities, particularly trochlear dysplasia, only with extrarticular procedure and with a low complication rate.

## Materials and methods

The reported study was a prospective observational longitudinal study. During the period between January 2014 and May 2019, 25 patients with patellar instability were studied after undergoing a surgical procedure combining the lowering/transposition of the ATT and the reconstruction of the MPFL, with a follow-up after 3, 6 and 12 months.

Patients signed the institutional consent for the use of their data for research purpose, as approved by the local Ethical Committee. It was a written informed consent document.

The criteria of inclusion were:

- symptomatic patient with at least 2 episodes of patellar dislocation
- distress or actual injury of the MPFL related to the dislocations themselves

The criteria of exclusion were:

- focal chondral lesion
- isolated injuries of the MPFL without others risk factors for patellar instability
- patella alta without MPFL insufficiency
- associated injuries of the cruciate and collateral ligaments

Each patient’s pre-operative symptomatology and state was evaluated by the following scales: Kujala score, for anterior knee pain [19]; IKDC (International Knee Documentation Committee) subjective knee evaluation form, for symptoms, sports activity and joint function [16]; Tegner activity level scale, for sports activity [27, 29]. The above evaluation scales were re-submitted to patients 3, 6 and 12 months after surgery. The predisposing factors (patella alta and trochlear dysplasia) were evaluated by standard X-rays in lateral and axial projection with a 30° knee flexion; all data were assessed by the same radiologist. To measure the patellar height, the Caton–Deschamps index was calculated: an index above or equal to 1,4 was considered pathological. Moreover, computer tomography (CT) (“Siemens SOMATOM Sensation 64 slice CT Scanner”) was performed according to the Lyonnais protocol [28] for the evaluation of the TT-TG distance, sulcus angle and patellar tilt. MPFL injuries were studied by executing Magnetic Resonance Imaging (MRI) (“Siemens MAGNETOM Symphony 1.5 T”). Post-operative control was done by taking standard X-Ray in two projections, calculating the new Caton–Deshchamps index [12].

### Surgical technique

Based on diagnostic imaging, from which the patellar height and TT-TG distance in particular were determined, an osteotomy of the ATT was performed, with its consequent lowering and/or medialization, following the technique described by Fulkerson [15]. After standard preparation, each patient was placed in a supine position on the surgical table and, after evaluating the actual instability, the knee was kept flexed at 90°. A tourniquet was placed at the base of the limb. A paramedian skin incision with respect to the patellofemoral articulation was made, making it possible to approach the patellar tendon and the ATT after the dissection of the subcutaneous soft tissue. The lateral release of the retinaculum was always performed. The osteotomy was performed in such a way as to obtain a block of cortico-cancellous bone of about 80 mm, which includes the insertion point of the patellar tendon. The bone block thus obtained was mobilized and moved medially, according to the pre-operative planning, aiming to obtain a decrease of TT-TG index. The cut of the distal segment was made, and the remaining block was moved down to achieve the lowering of the ATT aiming to a Caton-Deschamps index (CDI) below 1.2. This repositioned ATT was secured with two bicortical screws (diameter 4,5 mm). After that we performed also MPFL reconstruction so as to add resistence to the forces that lateralize the patella. MPFL reconstruction was performed with autologous gracilis tendon or semitendinous tendon if gracis was insufficient.

**Fig. 1:**
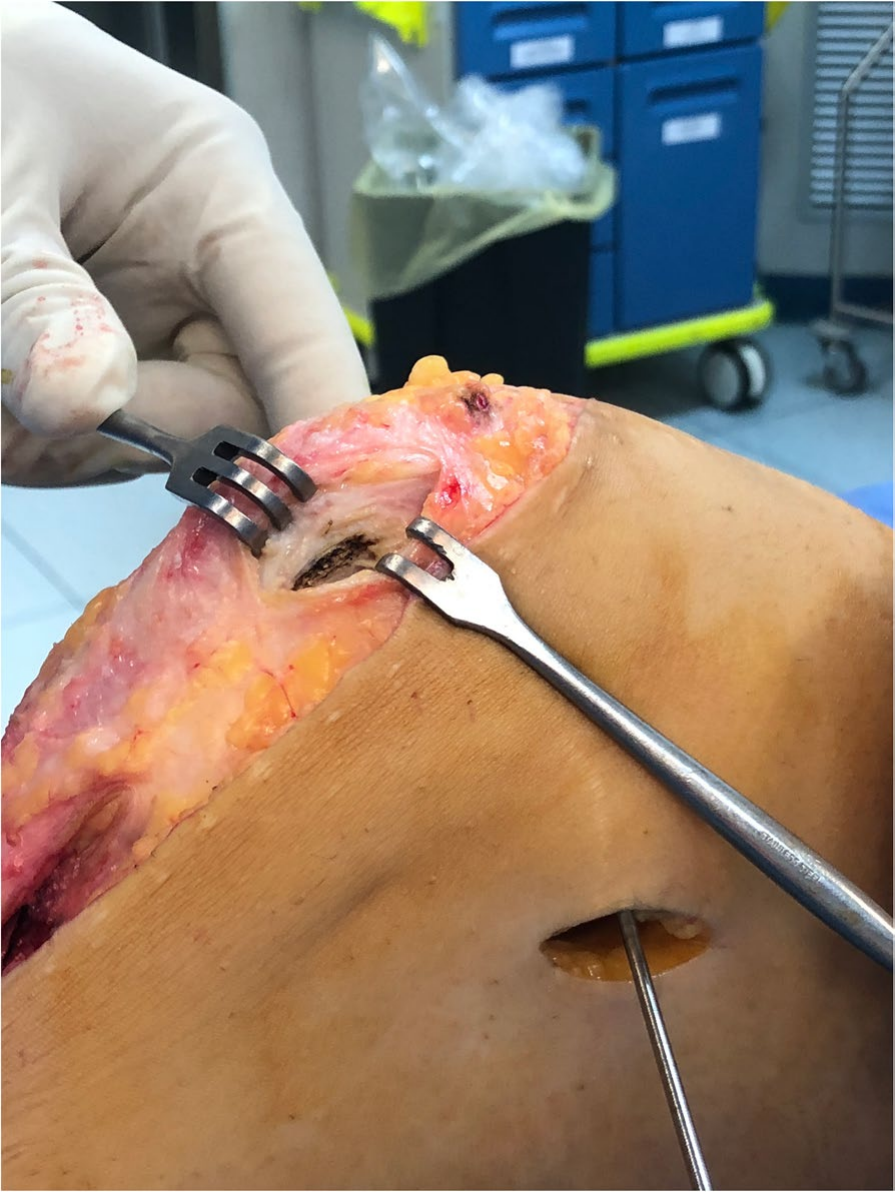
Gutter on the medial patellar head to obtain a full thickness subperiostal flap

**Fig. 2:**
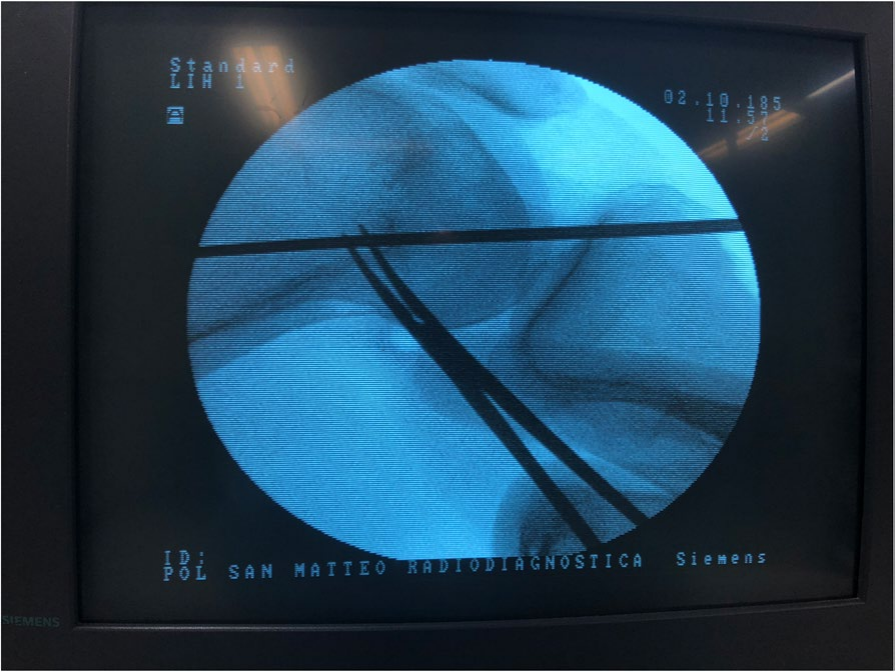
Identification of the femoral insertion point of the MPFL

**Fig. 3:**
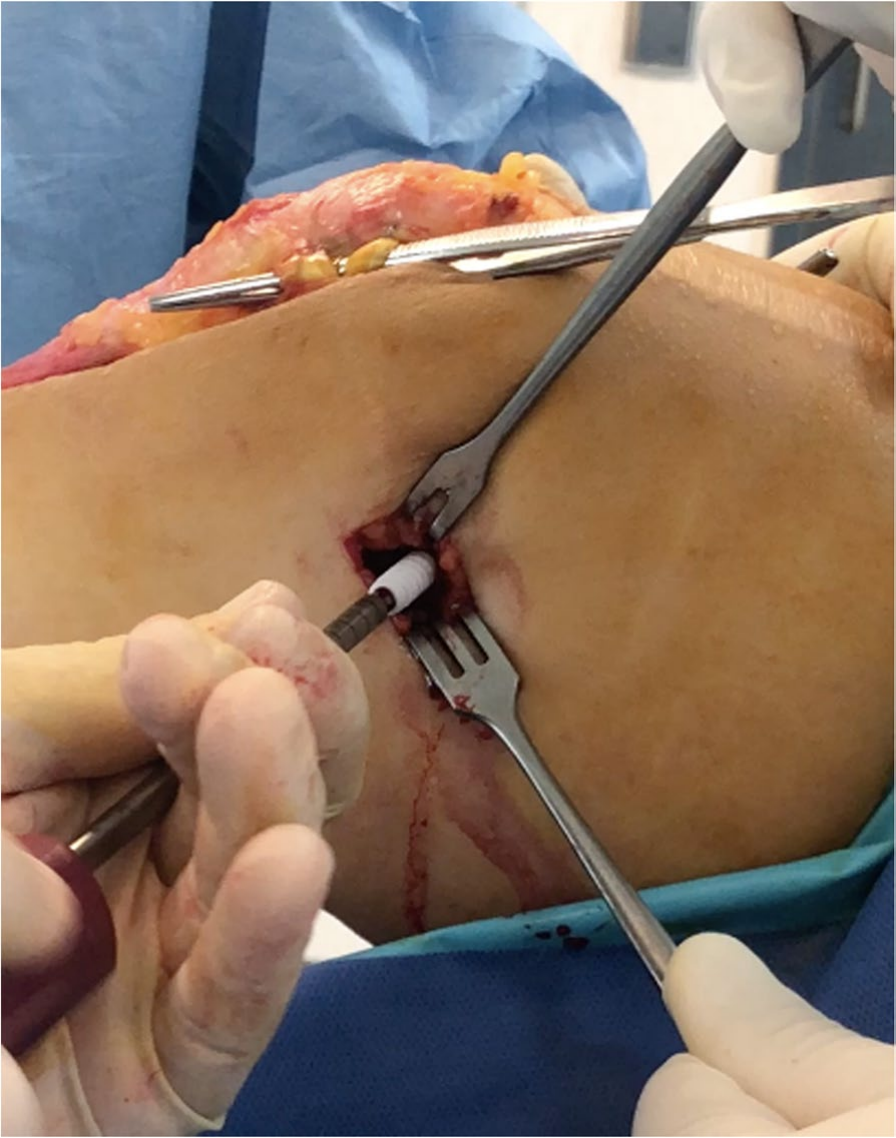
Fixation with an interference reabsorbable screw (7 × 28 mm) with the knee at 30° of flexion and neutral rotation

The graft was prepared using a reabsorbable suture (suture Ethicon Vicryl 1 J&J). Each of the two strands of the graft are stitched to reinforce and tubulize the extremities of the graft. A gutter was dug on the medial side of the patella. We have obtained a full thickness subperiostal flap (Fig. 1), which has included the original insertion point of the MPFL and the medial retinaculum. The gutter was dug in the proximal third of the patellar medial side [26], keeping it extra-articular. Under image intensifier control and by direct palpation, the adductor tubercle was identified as anatomical landmark (Fig. 2). A small skin incision is performed at this site. Under image intensifier the femoral insertion point of the MPFL, corresponding to the Schottle point [26], is identified. In correspondence to that spot, a femoral half tunnel (7X40 mm) was drill in the proximal-anterior direction over a guide wire. An extra-synovial soft tissue tunnel was then prepared in the second layer of the medial compartment that connects the medial part of the patella and the adductor tubercle. The sutured midpoint of the graft was placed in the tunnel dug in the patella and anchored with detached stitches to the periosteal flap of the patella. The gracilis tendon was then passed through the extra-synovial tunnel up to the femoral tunnel, where its extremity was fixed with an interference reabsorbable screw (7 × 28 mm) with the knee at 30° of flexion and neutral rotation (Fig. 3). This was necessary to ensure that the tension exerted by the graft was sufficient, but not excessive: the patella should be non-luxable, and the medial side should not be over-constrained. At this point, the tourniquet has been removed, a careful haemostasis has been performed, and drainage has been carried out. The skin has been sutured in layers. In the end, the knee has been put in a brace in extension.

### Rehabilitation

Post-operative rehabilitation aims to reduce the signs and symptoms of active inflammation, such as oedema and pain, as well as to restore an adequate range of motion (ROM) and adequate quadriceps strength. Adequate proprioception is also a target. Starting from the second post-operative day, active and passive mobilization were encouraged, progressing at 30° per week until complete recovery. Strengthening isometric quadriceps exercises with the brace in extension started from the second post-operative day. In the first three weeks, a tolerance weight bearing was allowed, using a brace that kept the knee in extension. Until the sixth week, the tolerance weight bearing was allowed with one or two crutches. Isotonic exercises to strengthen the femoral quadriceps muscle started from the sixth week. It was indicated, for example, to use low-resistance stationary bicycle, with a load that could be gradually increased compatibly with the pain. The return to sports activity was allowed starting from the fourth/fifth month.

### Statistical analysis

The calculations were made with the Stata 16 program (StataCorp, College Station, TX, USA). The continuous variables were described as average, standard deviation, or median and quartile, according to the distribution. Student’s test or Mann–Whitney’s U test was used to compare groups. The correlation was measured with Spearman’s R coefficient. The estimates were equipped with the corresponding 95% confidence interval. The categorical variables were described with numbers and percentages and compared to Fisher’s exact test. The variations in time were valued through linear regression models, generalized for repeated measurements. All tests were two-tailed. A *p* value < 0.05 was considered statistically significant.

## Results

The study examined 25 patients – 18 female (72%) and 7 male (28%) – who underwent MPFL reconstruction and ATT transposition for patellar instability and completed the proposed rehabilitation process. The average age of the patients was 20 years (range 13–43 years). Twelve patients underwent surgery on the right knee (48%) and 13 on the left knee (52%). The first episode of dislocation occurred following a traumatic event in 9 patients (36%); in the remaining 16 patients (64%), the first episode occurred spontaneously. No cases of re-dislocation occurred during the post-operative course; only one patient, 4 months after surgery, reported a traumatic fracture of the tibia as a complication after a sprain trauma. Trochlear dysplasia was found to be absent in 12 patients (48%) but present in 13 (52%). Nine patients were classified as grade A, according to the classification of Dejour [10, 11] (mild dysplasia), while 3 and 1 were, respectively, grade B and grade C (severe dysplasia) patients. The pre-operative CDI was normal / physiological (< 1.4) in 15 patients (60%) but pathological in 10 (40%). The sulcus angle was evaluated as elevated (> 144°) in 13 patients (52%) and normal in 12 (48%). Patellar tilt was considered abnormal (< 20°) in all patients (100%). The TT-TG distance was irregular (> 20 mm) in 17 patients (68%) and normal in 8 (32%)). No patient showed signs of patellar instability at the end of the follow-up. Table 1 shows the differentiation of the results from pre-operative to final follow-up at 12 months of the different scores used, and the pre- and post-surgery patellar height, which respectively show an increase in scores and a decrease in patellar height. There was no statistically significant correlation between the differential of Kujala score and IKDC for patients with or without trochlear dysplasia (*p* = 0.07 and *p* = 0.06). On the other hand, a statistically significant difference was demonstrated between the Lysholm score differential and patients with or without trochlear dysplasia (*p* = 0.03). However, no significant differences were found between any of Kujala differential, IKDC and Lysholm score and patients with or without pathological patellar height (*p* = 0.59; *p* = 0.57; *p* = 0.28), nor, again, between any of the three scores and patients with abnormal or less sulcus angle (*p* = 0.38; *p* = 0.53; *p* = 0.88). The correlation between the variation of the Kujala score and the patellar tilt was not statistically significant (Rho = -0.20; *p* = 0.33 (95% CI: -0.555 to 0.208)), as well as the correlation of the variation of the Kujala score with the TT-TG distance (Rho = 0.06; *p* = 0.76 (95% CI: -0.339 to 0.448)). Similar results were obtained by evaluating the correlation between the IKDC score and the patellar tilt (Rho = -0.35; *p* = 0.08 (95% CI: -0.660 to 0.043)), and the IKDC score and the TT-TG distance (Rho =—0.07; *p* = 0.73 (95% CI: -0.454 to 0.333)). On the other hand, there was a statistically significant correlation between the Lysholm score and patellar tilt (Rho = -0.40; *p* = 0.04 (95% CI: -0.687 to 0.006)). However, the correlation of Lysholm score with the TT-TG distance was not statistically significant (Rho = -0.23; *p* = 0.36 (95% CI: -0.575 to 0.179)). The correlation between the differential of the patella height and the patellar tilt was statistically significant (Rho = 0.41; *p* = 0.04 (95% CI: 0.018 to 0.693)), but the correlation of this same differential with the TT-TG distance was not statistically significant (Rho = 0.36).; *p* = 0.07 (95% CI: -0.032 to 0.666)). There was an increase in the scores of Kujala, IKDC and Lysholm after 3, 6 and 12 months, and the results were compared for the different follow-up times (Tables 2, 3, 4 and 5).

**Table 1:** Results from pre-operative to final follow-up at 12 months of the different scores used, and the pre- and post-surgery patellar height. (SD = standard deviation; Conf.inter. = confidence interval)

	Mean	SD	Min	P25	P50	P75	Max	95% conf.inter
KUJALA	39.16	21.41	2.00	23.00	44.00	55.00	78.00	30.32–47.99
IKDC	32.17	25.70	-17.20	11.50	41.40	47.30	70.30	21.56–42.77
LYSHOLM	44.12	18.25	8.00	30.00	46.00	57.00	70.00	36.58–51.65
PATELLA HEIGHT	0.44	0.26	0.00	0.30	0.40	0.60	1.10	0.330–0.541

**Table 2:**
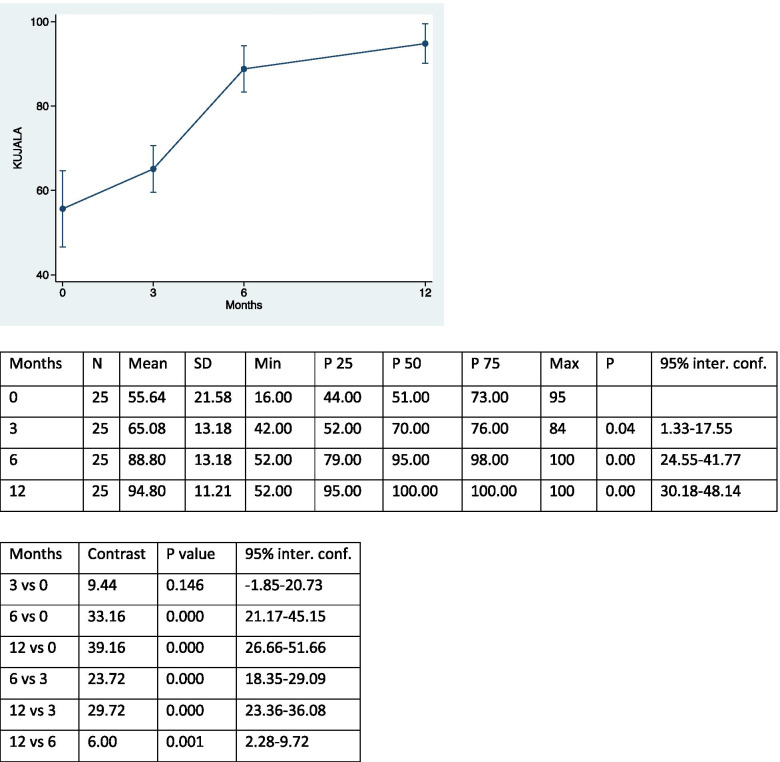
Kujala scores at the different follow up and comparison between each other

**Table 3:**
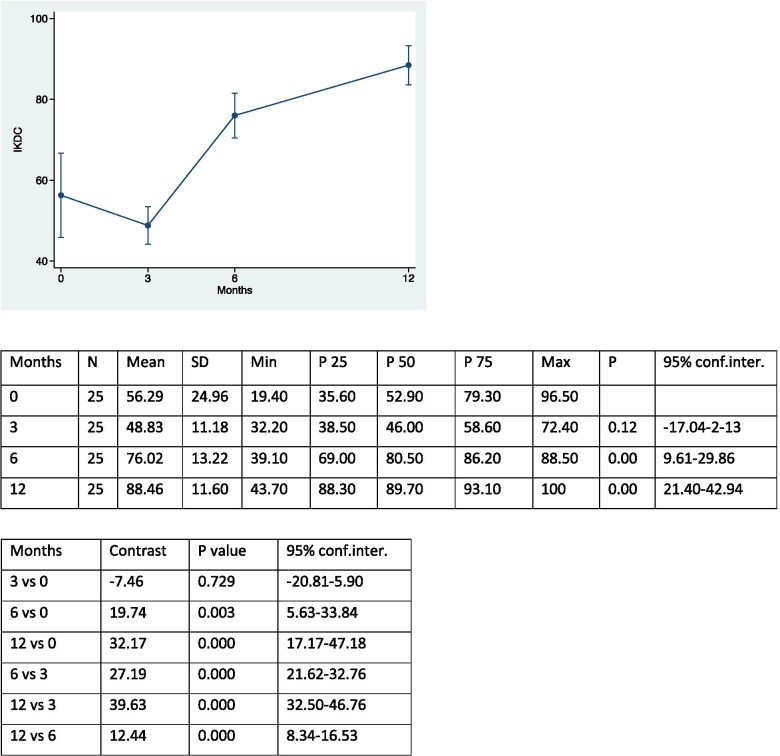
IKDC scores at the different follow up and comparison between each other

**Table 4:**
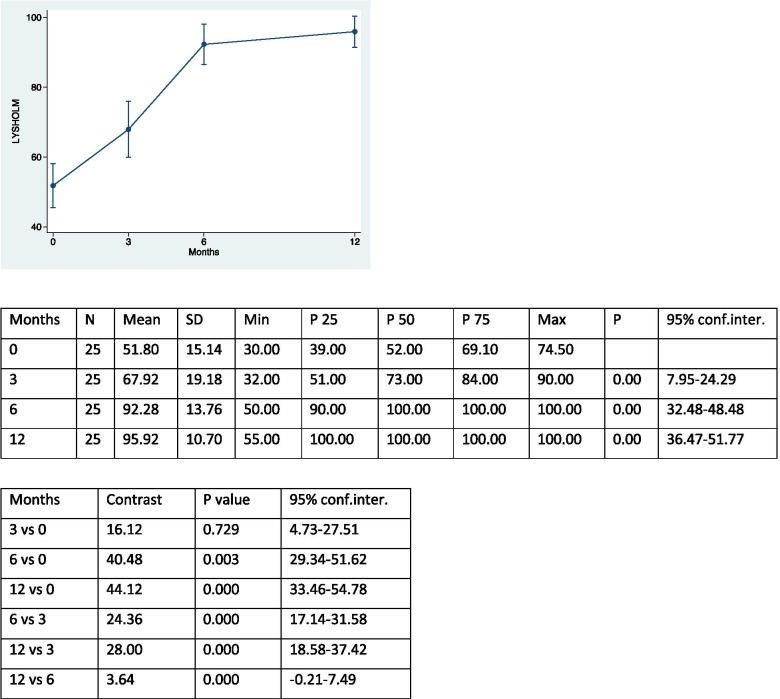
Lysholm scores at the different follow up and comparison between each other

**Table 5:** Values of Patellar Tilt (irregular > 20°), TT-TG (pathological > 20 mm) and preoperative and postoperative Patella height using Caton Deschamps Index (irregular > 1.4)

Patient	Patellar tilt	Tt-tg	Patella height preoperative	Patella height postoperative
1	47	21	1.6	0,93
2	26	24	1,3	1,06
3	27	21	1,23	1,09
4	27	21	1.36	1,08
5	35	24	2.13	1,03
6	25	14	1.4	1
7	24	14	1.24	1,02
8	25.9	21	1.30	1,04
9	32	14	1.6	1,01
10	23	16	1.28	0,98
11	25	22	1,6	0,82
12	24	20	1.23	1,01
13	26.2	15	1.33	1
14	29	22	1.8	1,2
15	27	18	1.36	0,98
16	24.3	9	1.65	1,1
17	24	22	1.37	1,1
18	26	25	2.01	1,5
19	29	20	1.7	1,36
20	28	27	1.25	0,73
21	26	24	1,6	1
22	23	20	2,1	1,5
23	23	15	1,2	1,1
24	20	20	1,08	1,1
25	26	22	1.08	1,06

The mean value of Patellar Tilt is 27.09 (range 20–47)

The mean value of TT-TG is 2 (range 0.9–2.7)

The mean value of Patellar Height preoperative is 1.44 (range 1.08–2.13)

The mean value of Patellar Height postperative is 1.07 (range 0.73–1.5)

TT-TG: tibial-tuberosity to trochlear groove distance

## Discussion

The excellent results and low incidence of recurrent dislocation and complications in patients treated with ATT transposition and MPFL reconstruction for patellar instability confirmed our initial thesis. The ATT transposition surgical procedure is currently the most common treatment for patellar dislocations [30]. However, the indications for the combined procedure are still under discussion in the literature. Recently, considerable attention has been focused on patients treated with isolated MPFL reconstruction, which is responsible for patellar medial stability in 50–60% [7]. However, in agreement with several authors, combining MPFL reconstruction with ATT transposition is recommended to prevent MPFL overload and achieve longer-lasting results, especially in patients with pathological TT-TG distance, high patella and trochlear dysplasia [5]. Wagner et al. reported low values ​​of the Kujala score in patients with high TT-TG distance that underwent MPFL reconstruction. A cadaver study reported that isolated MPFL reconstruction in patients with TT-TG distance > 10 mm would lead to increased graft tension and degenerative changes in the patella-femoral joint [2]. In our study, no new episodes of patellar instability were reported after surgery (0%), compared to a mean value of 36.4% according to a recent review in which all surgical procedures were still included. Similar rates were found in post-operative complications. In our study, an overall incidence of complications of 4% for tibial fracture after sprain trauma 4 months after surgery was reported. In the literature, the percentage was 6.5%, including superficial and deep infections, hematomas, deep vein thrombosis, post-traumatic arthrosis, paralysis of the peroneal nerve and paresis of the sciatic nerve [21]. Risk factors for patellar instability included in this study were trochlear dysplasia, sulcus angle, patellar tilt, patellar height and TT-TG distance. The patellar height, considered the most important risk factor for patellar instability, was calculated using the CDI, which is currently the most commonly used method for this purpose in the literature. A value equal to or greater than 1.4 indicates a severely high patella while a score between 0.6 and 1.2 defines a patella of normal height [20]. In our study, 40% of patients had a value equal to or greater than 1.4, in contrast to a recent review in which the proportion of patients was 18%. However, this low percentage was probably justified by the different exclusion criteria considered in the study. Recent biomechanical studies conducted by Watson et al. compared lateral restriction forces and patellofemoral contact pressure in patients with different patellar heights. They found that a higher patella reduced lateral restraining forces after MPFL reconstruction, thus demonstrating that a high patella may be a biomechanically severe risk factor for patellar dislocations [3]. The advantages of using the CDI is the ability to quantify changes in patellar height even after an osteotomy of the tibial tuberosity and, furthermore, to obtain measurements independently of the different degrees of flexion and different skeletal maturation. The main limitation, however, is the unclear identification of the patellar or tibial joint margin in arthritic knees [12]. The sulcus angle is a measurement that can be easily reproduced and is closely related to the other risk factors. For example, as the degree of trochlear dysplasia increases, the value of the sulcus angle also increases proportionally [8]. In our study, the sulcus angle was high (> 144°) in 52% of patients, the same percentage of patients with trochlear dysplasia. Among 13 patients, 9 had grade A of trochlear dysplasia, 3 grade B and 1 grade C, in accordance with Dejour's classification [9]. According to Allen et al., approximately 85% of patients with recurrent patellar instability have some degree of trochlear dysplasia [2]. Our lower percentages may be explained by the small number of patients examined in our work. Another parameter considered was the patellar tilt. It mainly corresponds to a dysplasia of the quadriceps muscle, in particular of the vastus medialis [13]. Patellar tilt was evaluated as pathological (> 20°) in all patients in the study. This is partly explained by the current literature, which has shown that an increase in values ​​can be a consequence of a high patella, trochlear dysplasia, increased TT-TG distance or insufficient MPFL [12]. A high value of the TT-TG distance, considered an important risk factor for lateral patellar instability, is not exclusive to a lateralization of the proximal tibia, but may be related to a medialization of the trochlear groove or to a rotational increase in the distal femur and proximal tibia [14]. It was high (> 20 mm) in our study in 68% of patients. However, anatomical variables play an important role in the measurement of TT-TG distance; for example, it is underestimated by up to 3 mm in patients with trochlear dysplasia compared to normal knees [12]. Finally, in agreement with the literature, we also found the female sex an important risk factor in patellar instability [6]. In fact, in our study, 72% of the patients enrolled in the study were female. Most of the scores used in the study reported an improvement in patient functionality and pain reduction in the various follow-ups. The increase by 39.13 points in the Kujala score from pre-operative values ​​to final follow-up after 12 months, compared to the 22.22 points presented by a recent review [5], suggests an important decrease in femoral patellar pain. A statistically significant increase in scores was obtained after 3, 6 and 12 months. Similar results were achieved by comparing the scores between the different follow-ups (12 vs 6, 12 vs 3, 6 vs 3, 12 vs 0, 6 vs 0), with the exception of the first interval of the first 3 post-operative months, where there was no statistically significant difference in the scores obtained. This result can be partly explained, in agreement with Thompson et al., by the adoption of a physiotherapy path which followed appropriate initial restrictions on the recovery of the range of motion and on the resumption of the load in order to reduce the risk of non-union or eventual detachment of the repositioned tuberosity, and which certainly limited the required activities such as running [30]. As with the Kujala score, a statistically significant increase in the IKDC rating scale was observed across the follow-ups. The presence, however, in the questionnaire of more demanding activities from a physical point of view (e.g. jumps and restarts) decreased the values, causing a reduction in scores in the first interval from 0 to 3 months. The Lysholm score, used initially for the evaluation of patients with anterior cruciate ligament injury, was subsequently shown to be more sensitive in the analysis of the functional disability of patients with reconstructed MPFL [5]. The scores obtained at the various follow-ups and compared with each other showed a statistically significant increase (except between 6 and 12 months). This data can be explained by the absence of high-demand activities in the questionnaire of the proposed score and by the presence of parameters that are normally reached after 6 months, such as the absence of swelling. Although the high patella is an important risk factor for patellar instability, no statistically significant differences were obtained for the different scores (Kujala, Lysholm and IKDC) between patients with patella height equal to or greater than 1.4 and patients with patella height < 1.4, which is in accordance with the report of Allen et al. [2], where the high patella had no direct relationship with the final results in patients treated with the combined procedure, but in contrast to results described by Sappey-Marinier et al. [25]. Another important risk factor for patellar dislocations is trochlear dysplasia. However, there is no agreement in the literature regarding the correlation between the presence and degree of dysplasia and clinical results. According to Hiemstra et al. [17], patellofemoral stabilization led to worse results in patients with a high degree of dysplasia, in contrast to Sappey-Marinier et al. [25], in which patients with a high degree of dysplasia showed no statistically significant differences in the Kujala scores. In our study, we found a significant difference between patients with and without trochlear dysplasia only in the Lysholm score. Patients with dysplasia presented a greater difference between pre-operative score and final follow-up than those without dysplasia, thus benefiting more from the operation. However, no significant differences were found for the Kujala and IKDC scores. The results obtained can be explained by the small number of participants, but also by the presence of a greater number of patients with low-grade trochlear dysplasia compared to high-grade ones. Therefore, the combination of MPFL reconstruction and ATT transposition in patients with trochlear dysplasia produce good clinical results and low degrees of re-dislocation, in agreement with the literature [18]. In our study, the pre-operative TT-TG distance did not affect the clinical results at the final and intermediate follow-ups, considering the disagreement in the literature regarding the measure being seen as pathological [1]. According to Matsushita et al. [23] no statistically significant difference in clinical scores was obtained between patients with a TT-TG distance > 20 mm and those with a TT-TG distance < 20 mm who underwent surgery for patellar instability. Schottle et al. [26] analyzed 15 patellar dislocation; 7 patients underwent only MPFL reconstruction, while 8 patients underwent MPFL reconstruction and ATT transposition, for cases with TT-TG distance > 15 mm. The two patient groups did not show statistically significant differences in the final results. Patients with high patellar tilt values ​​had a statistically significant greater increase in score only for the Lysholm score but not for the IKDC and Kujala. This data can again be explained by the absence of high-demand activities in the proposed score questionnaire with respect to Kujala and IKDC. Furthermore, patients whose patellar tilt was corrected more (thanks to a more decisive lateral-release) obtained a more significant improvement in functional scores, in accordance with Longo et al. where the combined procedures allowed a greater centring of the patella during movement, thus avoiding long-term complications [22]. Finally, a statistically significant correlation was found between the differential of the patella height and the patellar tilt. This can be partly explained because with the lowering of the ATT, there is still a minimal part of its medialization [27]. The surgical technique with lowering and medialization of the ATT makes it possible to obtain excellent functional results without new episodes of dislocation, thus improving the TT-TG distance. The limitations of the study are the limited number of patients and the short follow-up considered.

## Conclusion

This prospective observational longitudinal study identified good clinical outcomes in patients who underwent MPFL reconstruction and ATT transposition for patellar instability. Furthermore, no patient reported a new episode of patellar dislocation, demonstrating the safety of the surgical procedure. Only 1 patient reported a post-traumatic tibial fracture following a sprain trauma 4 months after surgery. Finally, the different risk factors for patellar instability examined, particularly the presence of trochlear dysplasia, did not significantly influence the final functional results, which were very satisfactory without re-dislocation episodes.
